# Postoperative Supplemental Oxygen in Liver Transplantation (PSOLT) does not reduce the rate of infections: results of a randomized controlled trial

**DOI:** 10.1186/s12916-023-02741-w

**Published:** 2023-02-13

**Authors:** Wojciech Figiel, Grzegorz Niewiński, Michał Grąt, Marek Krawczyk, Jan Stypułkowski, Zbigniew Lewandowski, Maciej Krasnodębski, Waldemar Patkowski, Krzysztof Zieniewicz

**Affiliations:** 1 Department of General, Transplant and Liver Surgery, Medical University of Warsaw, Banacha 1A, 02-097 Warsaw, Poland; 2 Department of Epidemiology and Biostatistics, Medical University of Warsaw, Oczki 3, 02-007 Warsaw, Poland

**Keywords:** Liver transplantation, Oxygen, Infection, Morbidity, Mortality, Ischemia-reperfusion injury

## Abstract

**Background:**

Despite inconsistent evidence, international guidelines underline the importance of perioperative hyperoxygenation in prevention of postoperative infections. Further, data on safety and efficacy of this method in liver transplant setting are lacking. The aim was to evaluate efficacy and safety of postoperative hyperoxygenation in prophylaxis of infections after liver transplantation.

**Methods:**

In this randomized controlled trial, patients undergoing liver transplantation were randomly assigned to either 28% or 80% fraction of inspired oxygen (FiO_2_) for 6 postoperative hours. Infections occurring during 30-day post-transplant period were the primary outcome measure. Secondary outcome measures included 90-day mortality, 90-day severe morbidity, 30-day pulmonary complications, durations of hospital and intensive care unit stay, and 5-day postoperative bilirubin concentration, alanine and aspartate transaminase activity, and international normalized ratio (INR) (clinicatrials.gov NCT02857855).

**Results:**

A total of 193 patients were included and randomized to 28% (*n* = 99) and 80% (*n* = 94) FiO_2_. With similar patient, operative, and donor characteristics in both groups, infections occurred in 34.0% (32/94) of patients assigned to 80% FiO_2_ as compared to 23.2% (23/99) of patients assigned to 28% FiO_2_ (*p* = 0.112). Patients randomized to 80% FiO_2_ more frequently developed severe complications (*p* = 0.035), stayed longer in the intensive care unit (*p* = 0.033), and had higher bilirubin concentration over first 5 post-transplant days (*p* = 0.043). No significant differences were found regarding mortality, duration of hospital stay, pulmonary complications, and 5-day aspartate and alanine transaminase activity and INR.

**Conclusions:**

Postoperative hyperoxygenation should not be used for prophylaxis of infections after liver transplantation due to the lack of efficacy.

**Trial registration:**

ClinicalTrials.gov NCT02857855. Registered 7 July 2016.

**Supplementary Information:**

The online version contains supplementary material available at 10.1186/s12916-023-02741-w.

## Background

Infections in patients undergoing surgery remain an important and global public health issue due to their incidence, impact on treatment outcomes, and associated costs [1, 2]. For prevention purposes, the World Health Organization (WHO) created international practice guidelines for perioperative period [3–5]. These include provision of 80% fraction of inspired oxygen (FiO_2_) for 2–6 h postoperatively to all adults. Similar recommendation was made by the Centers for Disease Control and Prevention (CDC) [6].

The rationale behind hyperoxygenation in prevention of infections is based on increased tissue oxygen tension facilitating the process of oxidative killing by neutrophils and thus augmenting immune response [7, 8]. This inexpensive and easy prophylactic measure was first found effective in reducing surgical site infections (SSIs) rate after colorectal procedures, with relative risk reduction exceeding 50% [9]. Subsequent studies, differing in details of oxygen administration and types of surgery, brought inconsistent results [10–15]. These were addressed by numerous meta-analyses pointing towards evidence for efficacy in all patients, evidence for efficacy in certain high-risk populations, or no evidence for efficacy [16–21]. This, in addition to the potential harms of hyperoxygenation, underlay the common criticism of the WHO and CDC recommendations [22–24].

Early postoperative infections are of particular concern in transplantation, due to the necessity of immunosuppression. Nearly half of liver transplantation (LT) recipients develop infections within 6 months after the procedure, with survival being influenced particularly by SSIs and number of infectious episodes [25]. Considering the previously reported benefits of high FiO_2_ in patients undergoing higher risk colorectal procedures and complete lack of data regarding patients undergoing LT, the latter seem of particular interest in evaluating the effects of perioperative hyperoxygenation. Further, increased formation of reactive oxygen species (ROS) secondary to hyperoxia may not only facilitate elimination of pathogens, but may also exacerbate the process of oxidative stress [26]. In LT, this may translate into higher degree of ischemia-reperfusion injury with potential negative effects on clinical outcomes [27, 28]. The aim of this study was to evaluate the efficacy and safety of providing high FiO_2_ to LT recipients in the immediate post-transplant period with respect to prevention of postoperative infections and allograft function, respectively.

## Methods

This was a randomized parallel controlled trial. Adult patients undergoing LT were screened for eligibility. Active status on the waiting list was the only inclusion criterion. Exclusion criteria comprised active infection, malignancy, cardiac arrest during transplantation, chronic obstructive pulmonary disease, and acute myocardial infarction. Recruitment took place between July 27, 2016, and March 2020 with follow-up for short-term outcomes completed on June 2020. The study protocol was approved by the institutional review board of the Medical University of Warsaw (KB/158/2016) and registered at ClinicalTrials.gov (NCT02857855) on July 7, 2016. All patients provided informed consent prior to inclusion.

Immediately after transplantation, patients were randomized by physician on duty in the department with a 1:1 allocation ratio, randomly selected blocks of 6, 8, and 10, and stratification by Child-Turcotte-Pugh class, to either 80% or 28% FiO_2_ for 6 postoperative hours. Random assignment was performed by drawing a sealed envelope with computer-generated code. Patients assigned to 80% FiO_2_ received 14 L/minute of oxygen and 2 L/minute of air through a non-rebreathing face mask with reservoir. Appropriate Venturi mask was used for patients assigned to 28% FiO_2_. In case of mechanical ventilation continued in the postoperative period, either 80% or 28% FiO_2_ was selected. Irrespective of the assignment, FiO_2_ could be increased in order to ensure arterial oxygen saturation > 92%. Patients and outcome assessors were unaware of the assignment.

The primary outcome measure was the occurrence of infection in the 30-day postoperative period according to CDC definitions [29]. Secondary outcome measures included 90-day postoperative mortality, 90-day severe postoperative morbidity (Clavien-Dindo grade ≥ 3), duration of postoperative hospital and intensive care unit stay, 30-day pulmonary complications, early allograft dysfunction (EAD) according to Olthoff et al., and 5-day serum bilirubin concentration, serum aspartate (AST) and alanine (ALT) transaminase activities, and international normalized ratio (INR) [30, 31]. Additionally, graft function was quantified using model for early allograft function (MEAF) and liver graft assessment following transplantation (L-GrAFT) models [32, 33]. Study on the Efficacy of Nosocomial Infection Control (SENIC) and National Nosocomial Infections Surveillance System (NNISS) risk scores were used to assess the initial SSI risk [34, 35]. This is a pre-planned analysis of the early outcomes (up to 90 days). Data on 5-year survival outcomes and biliary complications are yet to be collected.

The hypothesis was that provision of 80% FiO_2_ reduces the risk of postoperative infections from 40% to 24% (40% relative risk reduction, basing on Belda et al. study [10]). Sample size calculation based on thresholds for type I and type II errors of 0.05 and 0.20 with anticipated drop-out rate of 10% resulted in 296 patients (148 patients in each group). The study was prematurely terminated with the outbreak of COVID-19 pandemic in March 2020.

All patients received antibiotic prophylaxis, including intravenous piperacillin/tazobactam 4.5 g every 8 h for 3 days after first transplantations and meropenem every 8 h with vancomycin 1.0 g every 12 h adjusted for serum concentration for 7 days after re-transplantations. Antifungal prophylaxis comprised fluconazole 0.4 g every 24 h for 3 and 7 days, respectively. Hair was removed with a clipper immediately before patient transfer to the operating theater. Skin was prepared with povidone-iodine solution. Standard intraoperative FiO_2_ was 50%. Perioperative warming devices were used to avoid hypothermia. Continuous insulin infusion was applied in all patients for blood glucose control. Plastic adhesive drapes were routinely applied before skin incision. After fascial closure, aqueous povidone-iodine solution was used for wound irrigation. Three closed-suction drains were routinely left in the abdominal cavity and removed when clinically indicated. Skin was either stapled or closed by interrupted sutures. Conventional sterile dressing was changed and the wound examined daily. All patients were operated under laminar airflow. On first postoperative day, a routine abdominal ultrasonography was performed with additional computed tomography performed before discharge. Postoperative fluids included infusion of crystalloids at the standard rate of 1.5 ml/kg/h. All procedures were deceased donor LTs utilizing entire organs procured from donors after brain death.

Analyses were per intention-to-treat principle. Qualitative and quantitative variables were presented as *n* (%) and median (interquartile range [IQR]) or mean (standard error [SE]), respectively. Fisher’s exact test and Mann-Whitney *U* test were used for comparisons, as appropriate. Logistic regression was used for calculating odds ratios (ORs) with 95% confidence intervals (95%CIs). Mixed models were applied for longitudinal comparisons. For this purpose, natural logarithms of serum bilirubin concentration and transaminases activity were applied. Kaplan-Meier estimates were applied to estimate proportions of patients remaining in the intensive care unit (ICU) and in the hospital over time with log-rank test used for comparisons. The level of significance was set at two-tailed alpha level of 0.05. SAS/STAT version 15.2 (SAS Institute Inc., USA) was used for processing of statistical analyses.

## Results

**Fig. 1:**
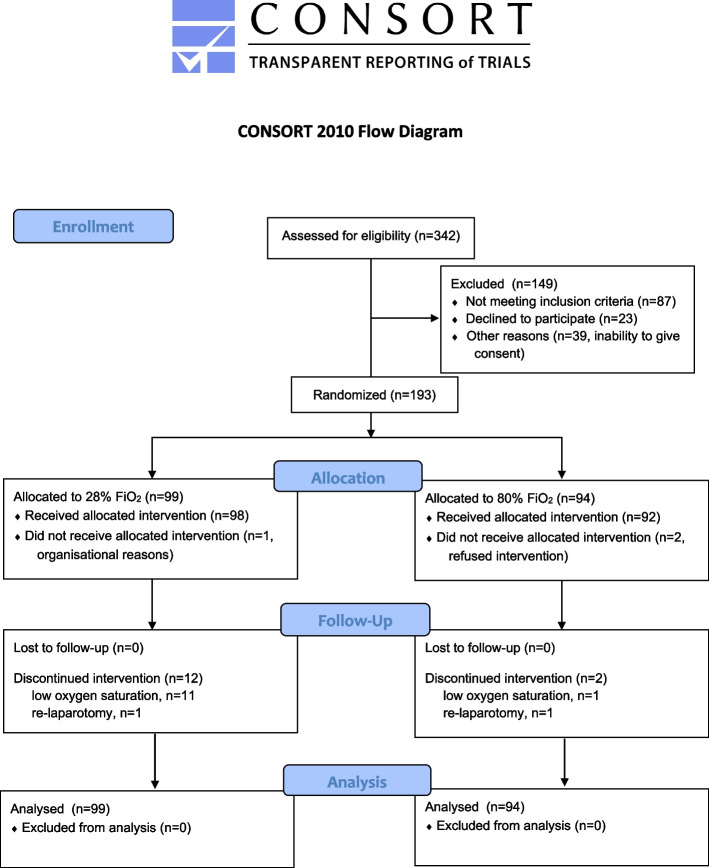
CONSORT flow diagram

A total of 193 patients were included and randomized into 80% (*n* = 94) and 28% (*n* = 99) FiO_2_ group (Fig. 1). Both groups were similar regarding baseline recipient, donor, and operative characteristics (Tables 1 and 2). Postoperative tacrolimus trough levels were similar in both groups (see Additional file 1: Fig. S1). No patient was in the ICU prior to transplantation. At the end of intervention, patients assigned to 80% FiO_2_ had significantly higher median PaO_2_ (*p* < 0.001) and oxygen saturation (*p* < 0.001) with no differences in arterial pH, PaCO_2_, and lactate concentration as compared to those assigned to 28% FiO_2_. Postoperative infections occurred in 55 patients (28.5%), including 37 SSIs, 11 urinary tract infections, 9 gastrointestinal infections, 8 pneumonias, 2 bloodstream infections, and 1 vascular access infection. Most frequent SSIs were associated with prolonged ICU and hospital stay, similarly to other infections (see Additional file 2: Fig. S2). Two of patients with SSI developed sepsis (1 died) and further 3 required abscess drainage (1 percutaneous; 2 operative).

**Table 1:** Comparison of baseline recipient data between patients undergoing liver transplantation assigned to 28% and 80% postoperative FiO_2_

Variables	28% FiO_**2**_ (***n*** = 99)	80% FiO_**2**_ (***n*** = 94)	***p***
Patient age (years)	48.6 (39.0–58.9)	50.7 (40.3–58.6)	.673
Male sex	55 (55.6%)	59 (62.8%)	.380
Body mass index (kg/m^2^)	25.0 (22.9–27.7)	25.9 (23.2–28.3)	.360
MELD	14 (11–18)	14 (10–20)	.739
Child-Turcotte-Pugh class:			.962
A	37 (37.4%)	34 (36.2%)	
B	46 (46.5%)	43 (45.7%)	
C	16 (16.2%)	17 (18.1%)	
Etiology of liver disease:			
Alcoholic liver disease	27 (27.3%)	29 (30.9%)	.636
HCV	14 (14.1%)	14 (14.9%)	> .999
HBV	6 (6.1%)	13 (13.8%)	.091
Primary sclerosing cholangitis	15 (15.2%)	13 (13.8%)	.840
Primary biliary cirrhosis	12 (12.1%)	14 (14.9%)	.675
Autoimmune hepatitis	16 (16.2%)	13 (13.8%)	.691
Non-alcoholic steatohepatitis	1 (1.0%)	1 (1.1%)	> .999
Wilson’s disease	3 (3.0%)	2 (2.1%)	> .999
Secondary biliary cirrhosis	0 (0.0%)	1 (1.1%)	.487
Unknown	4 (4.0%)	5 (5.3%)	.743
Smoking	7 (7.1%)	3 (3.2%)	.332
ASA classification ≥ 3	91 (91.9%)	89 (94.7%)	.569
Chronic diseases			
Diabetes	9 (9.1%)	15 (16.0%)	.191
Coronary artery disease	3 (3.0%)	3 (3.2%)	> .999
Hypertension	18 (18.2%)	17 (18.1%)	> .999
Ulcerative colitis	7 (7.1%)	4 (4.3%)	.538
Colonization with multidrug-resistant pathogens	9 (9.1%)	11 (11.7%)	.640
SENIC:			.615
2	74 (74.7%)	72 (76.6%)	
3	25 (25.3%)	21 (22.3%)	
4	0 (0.0%)	1 (1.1%)	
NNISS:			.435
0	6 (6.1%)	3 (3.2%)	
1	69 (69.7%)	62 (66.0%)	
2	24 (24.2%)	29 (30.8%)	
Preoperative laboratory parameters			
Hemoglobin (g/dL)	11.6 (10–13.1)	11.4 (9.7–13.1)	.509
White blood cell count (10^3^/mm^3^)	5.3 (3.6–7.1)	5.6 (3.8–7.8)	.552
Platelets (10^3^/mm^3^)	100 (63–168)	114 (60–173)	.856
Albumins (g/dL)	3.5 (3.0–4.0)	3.6 (3.1–4.0)	.784
Bilirubin (mg/dL)	2.5 (1.2–5.3)	2.0 (1.1–4.2)	.645
Creatinine (mg/dL)	0.9 (0.7–1.2)	0.9 (0.7–1.1)	.836
International normalized ratio	1.3 (1.1–1.5)	1.3 (1.2–1.6)	.550
C-reactive protein (mg/L)	7.8 (2.3–19.5)	8.5 (3.5–17.3)	.769

Data are presented as *n* (%) or median (interquartile range)

*FiO*_*2*_ Fraction of inspired oxygen, *MELD* Model for end-stage liver disease, *HCV* Hepatitis C virus, *HBV* Hepatitis B virus, *ASA* American Society of Anesthesiologists, *SENIC* Study on the Efficacy of Nosocomial Infection Control, *NNISS* National Nosocomial Infections Surveillance System

**Table 2:** Comparison of baseline perioperative and donor data between patients undergoing liver transplantation assigned to 28% and 80% postoperative FiO_2_

Variables	28% FiO_**2**_ (***n*** = 99)	80% FiO_**2**_ (***n*** = 94)	***p***
Retransplantation	2 (2.0%)	4 (4.3%)	.435
Caval anastomosis:			.675
Piggyback	87 (87.9%)	80 (85.1%)	
Conventional	12 (12.1%)	14 (14.9%)	
Veno-venous bypass	17 (17.2%)	16 (17.0%)	> .999
Biliary anastomosis			.692
Duct-to-duct	85 (85.9%)	78 (83.0%)	
Hepaticojejunostomy	14 (14.1%)	16 (17.0%)	
Operative time (minutes)	370 (330–420)	363 (330–430)	.591
Extubation			.450
Early	84 (84.8%)	75 (79.8%)	
Late	15 (15.2%)	19 (20.2%)	
Cold ischemic time (minutes)	480 (422–558)	480 (415–535)	.439
Warm ischemic time (minutes)	50 (42–64)	53 (45–64)	.337
Intraoperative PRBC transfusions (units)	3 (0–6)	4 (1–7)	.294
Intraoperative FFP transfusions (units)	4 (0–6)	4 (2–7)	.070
Intraoperative dialysis	3 (3.0%)	4 (4.3%)	.715
Donor age (years)	49 (37–58)	48 (38–59)	.966
Male donor sex	62 (62.6%)	55 (58.5%)	.659
Immunosuppressive treatment:			
Steroids	99 (100%)	94 (100%)	-
Tacrolimus	97 (98.0%)	90 (95.7%)	.435
Mycophenolate mofetil	49 (49.5%)	45 (47.9%)	.886
Basiliximab	72 (72.7%)	67 (71.3%)	.873
Laboratory parameters after intervention			
PaO_2_ (mmHg)	103 (89–122)	311 (255–364)	**< .001**
PaCO_2_ (mmHg)	41 (36–46)	41 (36–46)	.933
pH	7.38 (7.33–7.41)	7.36 (7.33–7.41)	.470
Oxygen saturation (%)	98.6 (97.5–99.0)	99.7 (99.5–99.9)	**< .001**
Lactate concentration (mmol/L)	1.8 (1.1–2.7)	1.9 (1.4–2.6)	.217

Data are presented as *n* (%) or median (interquartile range)

*FiO*_*2*_ Fraction of inspired oxygen, *PRBC* Packed red blood cells, *FFP* Fresh frozen plasma, *PaO*_*2*_ Arterial partial oxygen pressure, *PaCO*_*2*_ Arterial partial carbon dioxide pressure

A total of 32 (34.0%) patients randomized to 80% FiO_2_ developed postoperative infections in the 30-day post-transplant period as compared to 23 (23.2%; *p* = 0.112; Table 3) patients randomized to 28% FiO_2_. No significant differences were observed between groups regarding the occurrence of particular types of infections. Adjusted for post-intervention oxygen saturation, assignment to 80% FiO_2_ was significantly associated with increased incidence of infections (*p* = 0.021). No other significant associations between high postoperative FiO_2_ and occurrence of infections were found in analyses adjusted for other covariates (see Additional file 3: Table S1). Comparisons of 30-day infection rates between patients assigned to 28% and 80% FiO_2_ in selected subgroups also revealed no significant differences (see Additional file 4: Table S2). Higher post-intervention oxygen saturation significantly decreased the risk of infections in patients assigned to 28% FiO_2_ (OR 0.79 per 1%, 95% CI 0.64–0.99; *p* = 0.040), contrary to those assigned to 80% FiO_2_ (OR 1.59 per 1%, 95% CI 0.62–4.10; *p* = 0.339). Arterial partial oxygen pressure was neither associated with infections in the 28% FiO_2_ group (OR 0.92 per 10 mmHg, 95% CI 0.78–1.08; *p* = 0.305) nor in 80% FiO_2_ group (OR 1.01 per 10 mmHg, 95% CI 0.96–1.07; *p* = 0.640).

**Table 3:** Comparisons of patients assigned to 80% and 28% fraction of inspired oxygen during 6 postoperative hours with respect to primary and secondary pre-specified and not pre-specified outcome measures

Outcome measures	28% FiO_**2**_ (***n*** = 99)	80% FiO_**2**_ (***n*** = 94)	***p***
Primary outcome measure			
30-day postoperative infections	23 (23.2%)	32 (34.0%)	.112
Surgical site infections	15 (15.2%)	22 (23.4%)	.200
Urinary tract infections	4 (4.0%)	7 (7.4%)	.363
Gastrointestinal infections	3 (3.0%)	6 (6.4%)	.321
Pneumonia	2 (2.0%)	6 (6.4%)	.161
Bloodstream infections	1 (1.0%)	1 (1.1%)	> .999
Cardiovascular system infections	0 (0.0%)	1 (1.1%)	.487
Other	0 (0.0%)	2 (2.1%)	.236
Secondary outcome measures			
90-day patient mortality rate	5 (5.1%)	4 (4.3%)	> .999
90-day severe morbidity rate	28 (28.3%)	41 (43.6%)	**.035**
Early allograft dysfunction	30 (30.3%)	36 (38.3%)	.288
Post-transplant hospital stay (days)	17.5 (0.7)	19.1 (0.7)	.113
Post-transplant ICU stay (days)	5.4 (0.2)	6.6 (0.4)	**.033**
30-day pulmonary complication rate	25 (25.3%)	29 (30.9%)	.425
Other (not pre-specified)			
Comprehensive complication index^a^	22.6 (8.7–45.7)	24.2 (12.2–48.1)	.060
Post-reperfusion syndrome^b^	22 (22.2%)	30 (31.9%)	.146
Acute kidney injury^c^	46 (46.5%)	54 (57.4%)	.150
Need for pressure support	11 (11.1%)	10 (10.6%)	> .999

Data are presented and *n* (%) or mean (standard error)

*FiO*_*2*_ Fraction of inspired oxygen, *ICU* Intensive care unit

^a^Calculated according to Slankamenac et al. [36]

^b^Defined as decrease in mean arterial pressure by at least 30% within 5 minutes after reperfusion lasting for at least 1 min

^c^Defined as either increase in serum creatinine concentration by at least 0.3 mg/dL over 48 h or by at least 50% over 7 days

**Fig. 2:**
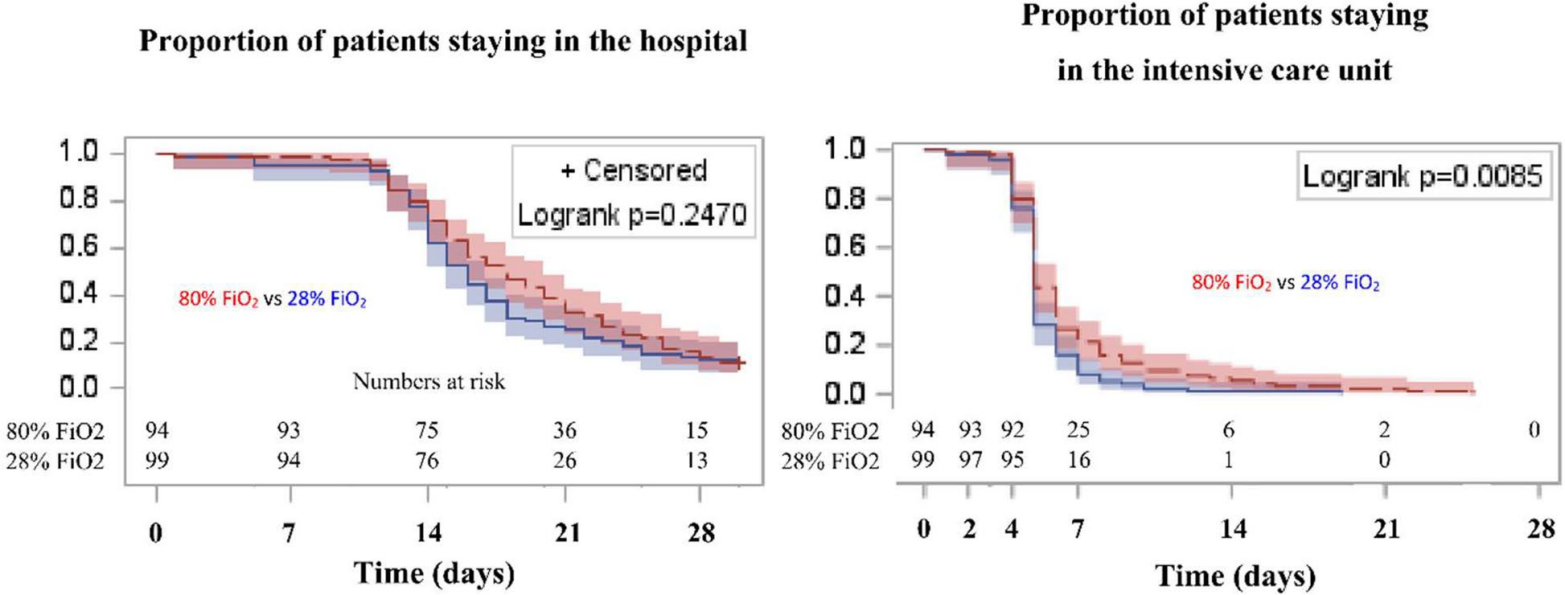
Proportion of patients staying in the hospital and staying in the intensive care unit after liver transplantation depending on the assignment to either 80% or 28% FiO_2_ for 6 postoperative hours. Red line represents patients assigned to 80% FiO_2_ and blue line represents patients assigned to 28% FiO_2_. Colored areas represent 95% confidence intervals

Patients receiving 80% FiO_2_ more frequently developed severe complications (*p* = 0.035; Table 3) and had longer ICU stay (*p* = 0.033). Particular types of severe complications in both groups are listed in Additional file 5, Table S3. The association between assignment to 80% FiO_2_ and higher morbidity rate was independent of the effects of patient, donor, and operative variables (see Additional file 6, Table S4). In two-factor analysis including FiO_2_ and post-intervention PaO_2_, the latter was a significant risk factor for severe morbidity (*p* = 0.010). No significant differences were found in patient mortality (*p* > 0.999), EAD rate (*p* = 0.288), pulmonary complication rate (*p* = 0.425), and duration of postoperative hospital stay (*p* = 0.113). Additionally, proportions of patients remaining in the ICU were significantly higher in the 80% FiO_2_ group (*p* = 0.009; Fig. 2).

**Fig. 3:**
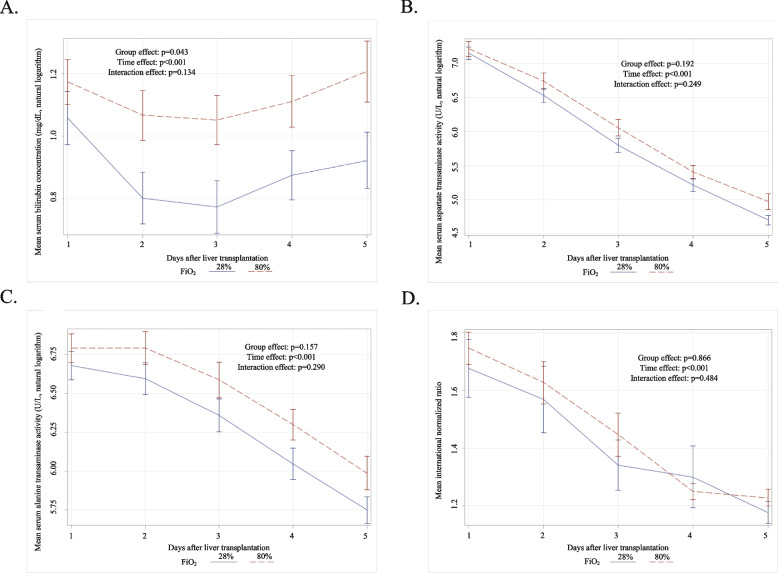
Mean serum bilirubin concentration (**A**), serum aspartate (**B**) and alanine (**C**) transaminase activity, and international normalized ratio (**D**) over first 5 days after liver transplantation in patients assigned to 80% (dashed lines) and 28% (solid lines) fraction of inspired oxygen for 6 postoperative hours. Vertical lines represent standard errors of the mean

Serum bilirubin concentration during first 5 post-transplant days was significantly higher in patients assigned to 80% FiO_2_ (*p* = 0.043; Fig. 3). There were no significant differences in INR (*p* = 0.866) and serum activity of aspartate (*p* = 0.192) and alanine aminotransferase (*p* = 0.157). Patients in high and low FiO_2_ had median MEAF of 6.34 (IQR: 5.19 to 7.71) and 6.15 (IQR: 4.97 to 7.67), respectively (*p* = 0.247), and corresponding median L-GrAFT of − 1.39 (IQR: − 2.90 to -0.42) and − 2.34 (IQR: − 2.98 to − 0.83), respectively (*p* = 0.068).

Of the 99 patients in the low oxygen group, 11 (11.1%) required FiO_2_ higher than 28% due to low oxygen saturation, 1 underwent re-laparotomy for bleeding after 2 h of intervention, and 1 received simple oxygen mask for organizational reasons. Of the patients requiring higher FiO_2_, 5 received oxygen concentration ≥ 60%. Of the 94 patients in high oxygen group, 1 required FiO_2_ ≥ 80%, 2 refused face mask, and 1 underwent re-laparotomy for bleeding after 4 h of intervention. Following exclusion of those patients in per-protocol analysis, infections occurred in 20 of 86 (23.3%) patients assigned to 28% FiO_2_ and in 30 of 90 (33.3%) patients assigned to 80% FiO_2_ (*p* = 0.181). High FiO_2_ remained significantly associated with severe morbidity with the corresponding rates of 25.6% (22/86) and 43.3% (39/90), respectively (*p* = 0.017). Proportion of patients remaining in the ICU after 7 and 14 days were 8.1% and 1.2%, respectively, in the 28% FiO_2_ group as compared to 20.0% and 4.4%, respectively, in the 80% FiO_2_ group (*p* = 0.032).

## Discussion

The use of high postoperative FiO_2_ failed to prevent postoperative infections in patients undergoing LT, both in general and with respect to the most common SSIs. Further, the current study did not provide evidence for the benefits of using high FiO2 regarding any other clinical outcomes. Therefore, in contrast to WHO and CDC recommendations, the results of this study do not support hyperoxygenation in the immediate postoperative period in LT recipients [4–6].

This findings are in contrast to the results of early randomized trials and the corresponding meta-analyses [9, 10, 37, 38]. Nevertheless, several subsequent studies, including the largest PROXI randomized trial performed on 1400 patients and alternating intervention trial including nearly 6000 patients, did not provide evidence for efficacy of perioperative hyperoxygenation in prophylaxis of postoperative infections [11, 13, 39–41]. In the updated meta-analysis, the evidence for the effectiveness of high perioperative FiO_2_ became weaker and limited to patients undergoing surgery under general anesthesia with endotracheal intubation [16]. The results of this study provide additional data supporting the need for revision of current WHO and CDC guidelines [4–6].

Irrespective of the previous studies focused on SSIs, this study provides first data on postoperative hyperoxygenation in LT recipients. Considering the high risk and wide spectrum of postoperative infections in this population, all infections occurring during the 30-day postoperative period were selected as the primary end-point, rather than only SSIs. However, no significant differences between study arms were observed also for particular types of infections, including SSIs, with the actual rates being higher in the 80% FiO_2_ group. Interestingly, higher oxygen saturation was protective against postoperative infections; however, this effect was limited to patients assigned to 28% FiO_2_. Further, adjusted for the effect of oxygen saturation in two-factor analysis, assignment to 80% FiO_2_ significantly increased the risk of infections. This suggests that while adequate oxygen delivery is important, hyperoxygenation does not seem to be an adequate method. In fact, it may cause significant vasoconstriction and paradoxically result in decreased delivery of oxygen to various tissues [42]. Therefore, while it seems that higher oxygen saturation reflecting adequate oxygen content is associated with fewer infections, the use of high FiO_2_ to further increase oxygen saturation does not provide further benefits.

Hyperoxygenation was reported to aggravate oxidative stress [26, 43, 44]. This seems to be responsible for the observed clinical outcomes, such as increased long-term mortality of cancer patients, shorter cancer-free survival, and increased mortality of acutely ill patients [45–47]. In this study, administration of 80% FiO_2_ was associated with prolonged hyperbilirubinemia, pointing towards the potential negative effect of hyperoxygenation on graft function. However, there were no significant differences in the quantitative measures of graft function, namely MEAF and L-GrAFT, and remaining laboratory measures. Therefore, the clinical relevance of prolonged hyperbilirubinemia associated with hyperoxygenation should be interpreted with caution.

In addition to the lack of effectiveness with respect to prophylaxis of infections, 80% FiO_2_ was associated with more frequent occurrence of severe complications and lower odds of early ICU discharge. Nevertheless, there were no significant differences in overall duration of hospital stay, potentially due to higher number of factors affecting it. Given similar patient, donor, and operative characteristics in both groups, this indicates that postoperative hyperoxygenation is not only ineffective but also potentially harmful in LT. While direct information on the causative mechanism cannot be drawn from the results, patients randomized to high FiO_2_ had non-significantly higher rates of EAD and pulmonary complications, non-significantly higher MEAF and L-GrAFT, and non-significantly higher INR and AST and ALT levels. Notably, the results of two-factor analysis of the association between FiO_2_ and PaO_2_ and the occurrence of severe complications pointed towards the negative effect of increasing PaO_2_ with no significant effect of FiO_2_. Conversely, more detailed comparison revealed that increased severe morbidity rate in the high FiO_2_ group was largely due to higher number of complications potentially caused by technical reasons, such as hemorrhage, hepatic artery thrombosis, and biliary leaks. Therefore, despite that severe complications were significantly more frequent in patients assigned to 80% FiO_2_, the current study does not prove the direct causative effect of high FiO_2_ on severe morbidity.

In this study, high FiO_2_ was applied for 6 h postoperatively with routine use of 50% intraoperative FiO_2_. While different duration of hyperoxygenation were used in previous studies, all were based on a combination of intraoperative and postoperative high FiO_2_. The most common duration of hyperoxygenation in the postoperative period was 2 h, with 6 h being the maximum recommended period. Considering that LT duration greatly exceeds that of the standard abdominal surgeries, hyperoxygenation limited to postoperative period was chosen for safety reasons and to provide uniform timing for all patients. Importantly, despite previous results and available recommendations, intraoperative 50% FiO_2_ seems to be the current standard [48].

The present study is subject to several limitations. First, the planned sample size was not reached due to study termination at the national outbreak of COVID-19 with the actual power of 66.5% for detection of the hypothesized 40% relative risk reduction from 40% to 24% with 80% FiO_2_. Nevertheless, the 30-day rate of postoperative infections, the primary outcome measure, was nearly 1.5 fold higher for the 80% FiO_2_ group. Therefore, type II error secondary to inadequate power does not seem to be a realistic bias. Second, a single-center design may limit generalizability of the findings. Nevertheless, it was associated with homogeneous epidemiological, prophylactic, and organizational approach. There were deviations from the assigned FiO_2_ in both groups, especially for patients randomized to 28% due to inadequate oxygen saturation. This, however, represents the real-world scenario and the results of per-protocol analyses were consistent with intention-to-treat findings. Further, administration of increased FiO_2_ to patients assigned to low FiO_2_ groups due to inadequate oxygen saturation was also common in other studies [9, 40, 41]. Although not all the guidelines regarding prevention of infections were followed (i.e., hair removal, prolonged antibiotic prophylaxis, lack of goal-directed fluid therapy) due to previously established center practices, this was uniform across study cohort.

## Conclusions

In conclusion, postoperative hyperoxygenation is ineffective in prevention of infections in LT recipients. Therefore, routine 80% postoperative FiO_2_ should be avoided in these patients.

## Supplementary Information

**Additional file 1: Figure S1.** Mean tacrolimus trough levels over 21 postoperative days in patients after liver transplantation assigned to 28% (solid line) and 80% (dashed line) fraction of inspired oxygen. Vertical lines represent standard errors of the mean.

**Additional file 2: Figure S2.** Proportion of patients staying in the hospital (A) and staying in the intensive care unit (ICU) (B) after liver transplantation depending development of surgical site infections (SSIs) and other infections. Colored areas represent 95% confidence intervals.

**Additional file 3: Table S1.** Effect of early postoperative fraction of inspired oxygen on infections after liver transplantation adjusted for subsequent covariates of interest in a series of two-factor analyses.

**Additional file 4: Table S2.** Subgroup analyses of 30-day infection rates after liver transplantation in patients assigned to 28% and 80% FiO2.

**Additional file 5: Table S3.** Severe morbidity in liver transplant recipients assigned to either 28% or 80% fraction of inspired oxygen for 6 postoperative hours.

**Additional file 6: Table S4.** Effect of early postoperative fraction of inspired oxygen on severe morbidity after liver transplantation adjusted for subsequent covariates of interest in a series of two-factor analyses.

## Data Availability

The datasets used and/or analyzed during the current study are available from the corresponding author on reasonable request.

## Abbreviations

95%CI: 95% confidence interval
ALT: Alanine transaminase
ASA: American Society of Anesthesiologists
AST: Aspartate transaminase
CDC: Centers for Disease Control and Prevention
EAD: Early allograft dysfunction
FFP: Fresh frozen plasma
FiO_2_: Fraction of inspired oxygen
HBV: Hepatitis B virus
HCV: Hepatitis C virus
ICU: Intensive care unit
INR: International normalized ratio
IQR: Interquartile range
L-GrAFT: Liver graft assessment following transplantation
LT: Liver transplantation
MEAF: Model for early allograft function
MELD: Model for end-stage liver disease
NNISS: National Nosocomial Infections Surveillance System
OR: Odds ratio
PaO_2_: Arterial partial oxygen pressure
PaCO_2_: Arterial partial carbon dioxide pressure
PRBC: Packed red blood cells
ROS: Reactive oxygen species
SE: Standard error
SENIC: Study on the Efficacy of Nosocomial Infection Control
SSI: Surgical site infection
WHO: World Health Organization
