# Supplementary material for: Postoperative Supplemental Oxygen in Liver Transplantation (PSOLT) does not reduce the rate of infections: results of a randomized controlled trial
Source: BMC Med. 2023 Feb 13;21:51. doi: 10.1186/s12916-023-02741-w (PMC9924861; doi:10.1186/s12916-023-02741-w)
Supplement: Supplementary file 1 — Additional file 1: Figure S1. Mean tacrolimus trough levels over 21 postoperative days in patients after liver transplantation assigned to 28% (solid line) and 80% (dashed line) fraction of inspired oxygen. Vertical lines represent standard errors of the mean. [file 12916_2023_2741_MOESM1_ESM.docx]

Figure S1. Mean tacrolimus trough levels over 21 postoperative days in patients after liver transplantation assigned to 28% (solid line) and 80% (dashed line) fraction of inspired oxygen. Vertical lines represent standard errors of the mean.


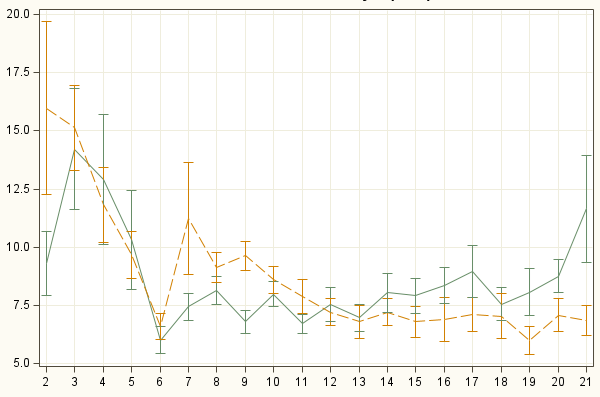


Group effect, p=0.943

Time effect, p<0.001

Interaction effect, p=0.031
