# Supplementary material for: Postoperative Supplemental Oxygen in Liver Transplantation (PSOLT) does not reduce the rate of infections: results of a randomized controlled trial
Source: BMC Med. 2023 Feb 13;21:51. doi: 10.1186/s12916-023-02741-w (PMC9924861; doi:10.1186/s12916-023-02741-w)

Figure S2. Proportion of patients staying in the hospital (A) and staying in the intensive care unit (ICU) (B) after liver transplantation depending development of surgical site infections (SSIs) and other infections. Colored areas represent 95% confidence intervals.

B.

A.


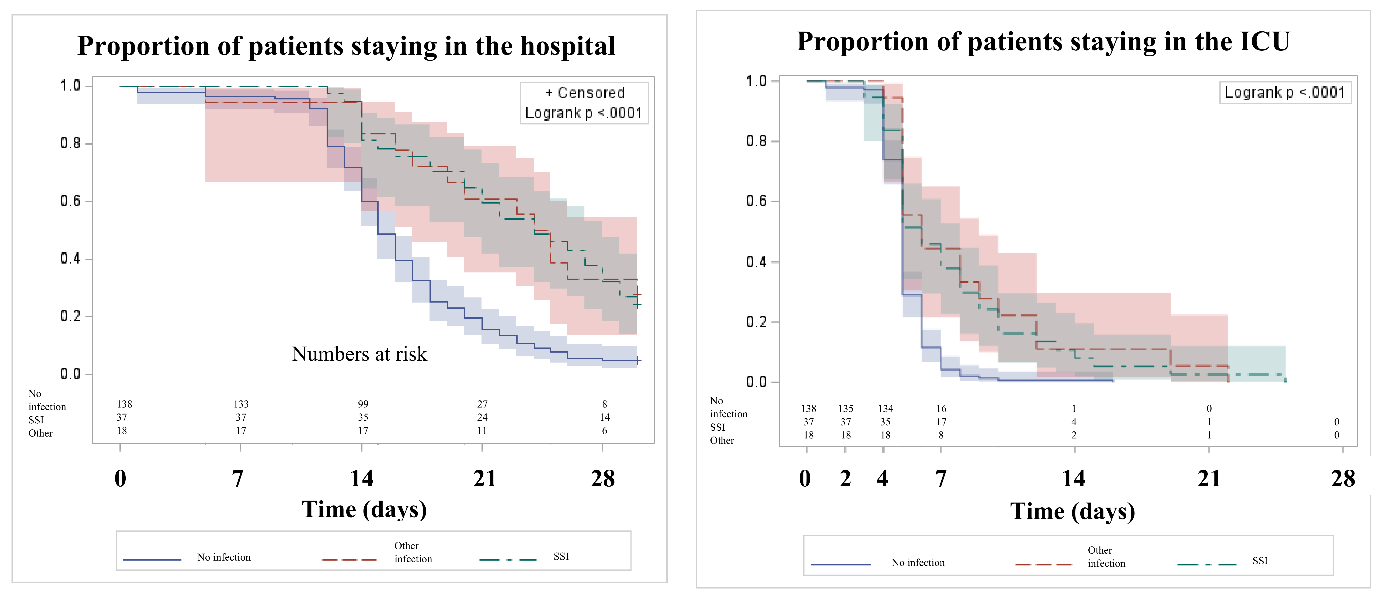

Supplement: Supplementary file 2 — Additional file 2: Figure S2. Proportion of patients staying in the hospital (A) and staying in the intensive care unit (ICU) (B) after liver transplantation depending development of surgical site infections (SSIs) and other infections. Colored areas represent 95% confidence intervals. [file 12916_2023_2741_MOESM2_ESM.docx]
