# Supplementary material for: Postoperative Supplemental Oxygen in Liver Transplantation (PSOLT) does not reduce the rate of infections: results of a randomized controlled trial
Source: BMC Med. 2023 Feb 13;21:51. doi: 10.1186/s12916-023-02741-w (PMC9924861; doi:10.1186/s12916-023-02741-w)
Supplement: Supplementary file 3 — Additional file 3: Table S1. Effect of early postoperative fraction of inspired oxygen on infections after liver transplantation adjusted for subsequent covariates of interest in a series of two-factor analyses. [file 12916_2023_2741_MOESM3_ESM.docx]

|  | | | | |
| --- | --- | --- | --- | --- |
| Table S1. Effect of early postoperative fraction of inspired oxygen on infections after liver transplantation adjusted for subsequent covariates of interest in a series of two-factor analyses. | | | | |
| **FiO_2_  80% versus 28%** | | **Covariate** | **OR (95% CI)** | **p** |
| **OR (95% CI)** | **p** |  |  |  |
| 1.70 (0.90 – 3.20) | .100 | Patient age | 1.06 (0.82 – 1.37) | .637 |
| 1.75 (0.93 – 3.30) | .085 | Male sex | 0.74 (0.39 – 1.40) | .349 |
| 1.78 (0.94 – 3.39) | .079 | Body mass index | 0.91 (0.84 – 0.99) | .031 |
| 1.72 (0.91 – 3.24) | .095 | MELD | 1.02 (0.98 – 1.06) | .298 |
| 1.70 (0.90 – 3.20) | .102 | Child-Turcotte-Pugh class: |  |  |
|  |  | A | Ref |  |
|  |  | B | 0.89 (0.44 – 1.80) | .735 |
|  |  | C | 1.44 (0.59 – 3.48) | .421 |
| 1.73 (0.92 – 3.25) | .092 | Alcoholic liver disease | 0.76 (0.37 – 1.55) | .449 |
| 1.72 (0.91 – 3.25) | .094 | HCV | 0.49 (0.18 – 1.38) | .176 |
| 1.66 (0.88 – 3.15) | .118 | HBV | 1.38 (0.51 – 3.76) | .530 |
| 1.70 (0.90 – 3.20) | .099 | Primary sclerosing cholangitis | 0.82 (0.33 – 2.07) | .678 |
| 1.70 (0.90 – 3.20) | .100 | Primary biliary cirrhosis | 1.10 (0.45 – 2.72) | .835 |
| 1.69 (0.90 – 3.19) | .106 | Autoimmune hepatitis | 0.48 (0.17 – 1.34) | .163 |
| 1.68 (0.89 – 3.17) | .108 | Smoking | 0.68 (0.14 – 3.34) | .632 |
| 1.68 (0.89 – 3.17) | .109 | ASA classification ≥3 | 2.19 (0.47 – 10.28) | .322 |
| 1.66 (0.88 – 3.14) | .119 | Diabetes | 1.49 (0.60 – 3.68) | .386 |
| 1.72 (0.91 – 3.25) | .094 | Coronary artery disease | –^a^ | .981 |
| 1.71 (0.91 – 3.23) | .097 | Hypertension | 0.57 (0.23 – 1.40) | .220 |
| 1.68 (0.89 – 3.17) | .107 | Ulcerative colitis | 0.58 (0.12 – 2.78) | .492 |
| 1.69 (0.90 – 3.19) | .103 | Colonization with multidrug-resistant pathogens | 1.36 (0.51 – 3.64) | .543 |
| 1.72 (0.91 – 3.25) | .094 | SENIC: |  |  |
|  |  | 2 | Ref |  |
|  |  | 3 | 0.63 (0.29 – 1.39) | .254 |
|  |  | 4 | –^a^ | .992 |
| 1.67 (0.88 – 3.15) | .115 | NNISS: |  |  |
|  |  | 0 | Ref |  |
|  |  | 1 | 3.07 (0.37 – 25.59) | .300 |
|  |  | 2 | 3.13 (0.36 – 27.42) | .303 |
| 1.68 (0.89 – 3.18) | .109 | Caval anastomosis: |  |  |
|  |  | piggyback | Ref |  |
|  |  | Conventional | 2.01 (0.85 – 4.74) | .111 |
| 1.73 (0.91 – 3.27) | .094 | Veno-venous bypass | 2.17 (0.99 – 4.75) | .053 |
| 1.68 (0.89 – 3.18) | .108 | Biliary anastomosis |  |  |
|  |  | duct-to-duct | Ref |  |
|  |  | hepaticojejunostomy | 1.82 (0.81 – 4.13) | .149 |
| 1.70 (0.90 – 3.21) | .100 | Operative time | 1.01 (0.77 – 1.33) | .926 |
|  |  | Preoperative laboratory parameters |  |  |
| 1.69 (0.90 – 3.19) | .104 | Hemoglobin | 0.93 (0.82 – 1.05) | .253 |
| 1.70 (0.90 – 3.20) | .100 | White blood cell count | 1.02 (0.92 – 1.13) | .714 |
| 1.68 (0.89 – 3.17) | .108 | Platelets | 1.02 (0.98 – 1.05) | .366 |
| 1.71 (0.91 – 3.22) | .096 | Albumins | 0.88 (0.57 – 1.37) | .574 |
| 1.67 (0.87 – 3.18) | .122 | Bilirubin | 1.06 (1.01 – 1.11) | .010 |
| 1.71 (0.91 – 3.22) | .097 | Creatinine | 0.94 (0.56 – 1.59) | .827 |
| 1.70 (0.91 – 3.21) | .099 | International normalized ratio | 0.99 (0.78 – 1.25) | .922 |
| 1.72 (0.91 – 3.24) | .095 | C-reactive protein | 1.02 (0.99 – 1.04) | .219 |
| 1.80 (0.95 – 3.42) | .073 | Cold ischemic time | 1.17 (1.00 – 1.37) | .052 |
| 1.75 (0.93 – 3.30) | .085 | Warm ischemic time | 1.04 (0.97 – 1.12) | .300 |
| 1.72 (0.91 – 3.25) | .094 | Intraoperative PRBC transfusions | 0.99 (0.91 – 1.07) | .768 |
| 1.69 (0.89 – 3.21) | .107 | Intraoperative FFP transfusions | 1.01 (0.93 – 1.09) | .894 |
| 1.73 (0.92 – 3.25) | .092 | Intraoperative dialysis | 0.39 (0.05 – 3.30) | .384 |
| 1.71 (0.90 – 3.22) | .099 | Donor age | 1.18 (0.93 – 1.50) | .166 |
| 1.68 (0.88 – 3.18) | .114 | Male donor sex | 0.47 (0.25 – 0.89) | .020 |
| 1.67 (0.89 – 3.16) | .112 | Retransplantation | 2.39 (0.46 – 12.41) | .299 |
| 1.68 (0.89 – 3.16) | .110 | Late extubation | 1.41 (0.64 – 3.12) | .391 |
|  |  | Immunosuppression |  |  |
| 1.72 (0.91 – 3.24) | .095 | basiliximab | 1.39 (0.67 – 2.86) | .380 |
| 1.67 (0.89 – 3.16) | .112 | tacrolimus | 0.42 (0.08 – 2.17) | .299 |
| 1.72 (0.91 – 3.24) | .094 | mycophenolate mofetil | 1.41 (0.75 – 2.65) | .290 |
|  |  | Laboratory parameters after intervention |  |  |
| 1.63 (0.52 – 5.08) | .402 | PaO_2_ | 1.00 (0.96 – 1.05) | .944 |
| 1.67 (0.88 – 3.17) | .117 | PaCO_2_ | 1.61 (1.05 – 2.47) | .030 |
| 1.71 (0.91 – 3.23) | .098 | pH | 1.00 (0.36 – 2.78) | .996 |
| 2.45 (1.15 – 5.24) | .021 | oxygen saturation | 0.83 (0.68 – 1.02) | .072 |
| 1.70 (0.90 – 3.21) | .101 | lactate concentration | 0.98 (0.88 – 1.09) | .678 |
| a – the estimates of coefficients were not presented due to the overestimation resulting from the small number of events. FiO_2_ – fraction of inspired oxygen; OR – odds ratio; 95% CI – 95% confidence interval; MELD – model for end-stage liver disease; HCV – hepatitis C virus; HBV – hepatitis B virus; ASA – American Society of Anesthesiologists; SENIC – Study on the Efficacy of Nosocomial Infection Control; NNISS – National Nosocomial Infections Surveillance System; PRBC – packed red blood cells; FFP – fresh frozen plasma; PaO_2_ – arterial partial oxygen pressure; PaCO_2_ – arterial partial carbon dioxide pressure. Odds ratios were calculated per 10 years increase for patient and donor age; 1 kg/m^2^ increase for BMI; 1 point increase for MELD; 1 hour increase for operative time and cold ischemic time; 1 g/dL increase for hemoglobin; 10^3^/cm^3^ increase for white blood count; 100 x 10^3^/cm^3^ increase for platelets; 1 g/dL increase for albumins; 1 mg/dL increase for bilirubin and creatinine; 1 increase for international normalized ratio; 1 mg/L increase for C-reactive protein; 10 min increase for warm ischemic time; 1 unit increase for PRBC and FFP transfusions; 10 mmHg increase for PaO_2_ and PaCO_2_; 1 increase for pH; 1% increase for oxygen saturation; 1 mmol/L increase for lactates. | | | | |
