# Supplementary material for: Postoperative Supplemental Oxygen in Liver Transplantation (PSOLT) does not reduce the rate of infections: results of a randomized controlled trial
Source: BMC Med. 2023 Feb 13;21:51. doi: 10.1186/s12916-023-02741-w (PMC9924861; doi:10.1186/s12916-023-02741-w)
Supplement: Supplementary file 4 — Additional file 4: Table S2. Subgroup analyses of 30-day infection rates after liver transplantation in patients assigned to 28% and 80% FiO2. [file 12916_2023_2741_MOESM4_ESM.docx]

| Table S2. Subgroup analyses of 30-day infection rates after liver transplantation in patients assigned to 28% and 80% FiO_2_. | | | |
| --- | --- | --- | --- |
| **Subgroup** | **30-day postoperative infection rate** | | **p** |
|  | **28% FiO_2_** | **80% FiO_2_** |  |
| MELD ≤14 | 24.0% (12/50) | 30.0% (15/50) | .653 |
| MELD >14 | 22.5% (11/49) | 38.6% (17/44) | .115 |
| Patients without technical complications^a^ | 20.5% (18/88) | 23.3% (17/73) | .704 |
| Patients without severe complications | 15.5% (11/71) | 17.0% ( 9/53) | >.999 |
| Patients with severe complications | 42.9% (12/28) | 56.1% (23/41) | .332 |
| Patients with early extubation | 22.6% (19/84) | 32.0% (24/75) | .213 |
| Patients with late extubation | 26.7% (4/15) | 42.1% (8/19) | .476 |
| a – including postoperative intraabdominal bleeding, stenosis of biliary anastomosis, biliary leak, hepatic artery kinking, large-for-size graft and burst abdomen  FiO_2_ – fraction of inspired oxygen; MELD – model for end-stage liver disease. | | | |
