# Supplementary material for: Postoperative Supplemental Oxygen in Liver Transplantation (PSOLT) does not reduce the rate of infections: results of a randomized controlled trial
Source: BMC Med. 2023 Feb 13;21:51. doi: 10.1186/s12916-023-02741-w (PMC9924861; doi:10.1186/s12916-023-02741-w)
Supplement: Supplementary file 5 — Additional file 5: Table S3. Severe morbidity in liver transplant recipients assigned to either 28% or 80% fraction of inspired oxygen for 6 postoperative hours. [file 12916_2023_2741_MOESM5_ESM.docx]

| Table S3. Severe morbidity in liver transplant recipients assigned to either 28% or 80% fraction of inspired oxygen for 6 postoperative hours. | | |
| --- | --- | --- |
| **Complication** | **28% FiO_2_**  **(n=99)** | **80% FiO_2_**  **(n=94)** |
| Grade III-V, any | 28^a^ | 41^a^ |
| Grade V (death) | 5 | 4 |
| Grade IV |  |  |
| Organ failure |  |  |
| Kidney | 6 | 5 |
| Respiratory | 8 | 7 |
| Circulatory | 8 | 10 |
| Liver |  |  |
| Primary non-function | 4 | 3 |
| Hepatic artery thrombosis | 1 | 5 |
| Other |  | 2 |
| Sepsis | 1 | 1 |
| Grade IIIB |  |  |
| Intraabdominal bleeding | 5 | 9 |
| Biliary leak | 5 | 10 |
| Biliary stenosis | 2 |  |
| Pleural bleeding |  | 1 |
| Burst abdomen |  | 1 |
| Large-for-size graft |  | 1 |
| Gastrointestinal bleeding | 1 | 1 |
| Portal thrombosis | 1 |  |
| Ileus | 1 |  |
| Incarcerated hernia |  | 1 |
| Grade IIIA |  |  |
| Pleural effusion | 6 | 8 |
| Hepatic artery kinking |  | 1 |
| Intraabdominal abscess |  | 1 |
| Non-infected intraabdominal collection |  | 1 |
| Wound hematoma |  | 1 |
| Non-infected incisional collection |  | 1 |
| Ascites | 2 |  |
| a – includes multiple severe complications in some patients | | |
