# Supplementary material for: Postoperative Supplemental Oxygen in Liver Transplantation (PSOLT) does not reduce the rate of infections: results of a randomized controlled trial
Source: BMC Med. 2023 Feb 13;21:51. doi: 10.1186/s12916-023-02741-w (PMC9924861; doi:10.1186/s12916-023-02741-w)
Supplement: Supplementary file 6 — Additional file 6: Table S4. Effect of early postoperative fraction of inspired oxygen on severe morbidity after liver transplantation adjusted for subsequent covariates of interest in a series of two-factor analyses. [file 12916_2023_2741_MOESM6_ESM.docx]

| Table S4. Effect of early postoperative fraction of inspired oxygen on severe morbidity after liver transplantation adjusted for subsequent covariates of interest in a series of two-factor analyses. | | | | |
| --- | --- | --- | --- | --- |
| **FiO_2_  80% versus 28%** | | **Covariate** | **OR (95% CI)** | **p** |
| **OR (95% CI)** | **p** |  |  |  |
| 2.00 (1.10-3.65) | .024 | Patient age | 0.84 (0.66-1.07) | .166 |
| 2.08 (1.13-3.82) | .018 | Male sex | 0.55 (0.30-1.01) | .052 |
| 2.01 (1.10-3.66) | .024 | Body mass index | 0.95 (0.88-1.02) | .158 |
| 1.97 (1.08-3.58) | .027 | MELD | 1.01 (0.98-1.05) | .550 |
| 1.96 (1.08-3.56) | .028 | Child-Turcotte-Pugh class: |  |  |
|  |  | A | Ref |  |
|  |  | B | 0.88 (0.45-1.70) | .691 |
|  |  | C | 1.10 (0.47-2.60) | .827 |
| 1.96 (1.08-3.56) | .028 | Alcoholic liver disease | 1.08 (0.56-2.08) | .811 |
| 1.97 (1.08-3.59) | .026 | HCV | 0.67 (0.28-1.63) | .375 |
| 2.02 (1.10-3.69) | .023 | HBV | 0.70 (0.25-1.96) | .490 |
| 1.96 (1.08-3.56) | .028 | Primary sclerosing cholangitis | 0.69 (0.28-1.67) | .410 |
| 1.94 (1.07-3.54) | .030 | Primary biliary cirrhosis | 1.61 (0.69-3.75) | .270 |
| 1.95 (1.07-3.55) | .029 | Autoimmune hepatitis | 0.66 (0.27-1.59) | .351 |
| 1.95 (1.07-3.56) | .029 | Smoking | 0.87 (0.21-3.53) | .840 |
| 1.94 (1.06-3.53) | .031 | ASA classification ≥3 | 1.82 (0.48-6.94) | .382 |
| 1.99 (1.09-3.64) | .025 | Diabetes | 0.79 (0.32-1.99) | .619 |
| 1.99 (1.09-3.64) | .025 | Coronary artery disease | –^a^ | .979 |
| 1.97 (1.08-3.58) | .027 | Hypertension | 0.67 (0.30-1.50) | .325 |
| 1.92 (1.05-3.51) | .034 | Ulcerative colitis | 0.18 (0.02-1.43) | .105 |
| 1.95 (1.07-3.56) | .028 | Colonization with multidrug-resistant  pathogens | 1.17 (0.45-3.07) | .744 |
| 1.99 (1.09-3.64) | .026 | SENIC: |  |  |
|  |  | 2 | Ref |  |
|  |  | 3 | 0.48 (0.22-1.03) | .059 |
|  |  | 4 | –^a^ | .991 |
| 1.98 (1.08-3.61) | .027 | NNISS: |  |  |
|  |  | 0 | Ref |  |
|  |  | 1 | 1.99 (0.39-10.15) | .406 |
|  |  | 2 | 1.44 (0.26-7.84) | .674 |
| 1.96 (1.08-3.56) | .028 | Caval anastomosis: |  |  |
|  |  | piggyback | Ref |  |
|  |  | conventional | 1.10 (0.47-2.61) | .824 |
| 1.96 (1.08-3.57) | .027 | Veno-venous bypass | 1.22 (0.56-2.65) | .624 |
| 1.97 (1.08-3.59) | .026 | Biliary anastomosis |  |  |
|  |  | duct-to-duct | Ref |  |
|  |  | hepaticojejunostomy | 0.85 (0.37-1.95) | .695 |
| 1.97 (1.08-3.58) | .027 | Operative time | 0.99 (0.76-1.28) | .915 |
| 1.94 (1.07-3.53) | .030 | Retransplantation | 1.65 (0.32-8.57) | .555 |
| 1.90 (1.03-3.51) | .039 | Late extubation | 3.12 (1.44-6.74) | .004 |
|  |  | Immunosuppression |  |  |
| 2.00 (1.10-3.67) | .024 | basiliximab | 1.93 (0.95-3.91) | .069 |
| 1.91 (1.03-3.53) | .040 | tacrolimus | –^a^ | .978 |
| 1.96 (1.08-3.56) | .027 | mycophenolate mofetil | 0.96 (0.53-1.74) | .882 |
|  |  | Preoperative laboratory parameters |  |  |
| 1.93 (1.06-3.52) | .031 | Hemoglobin | 0.98 (0.87-1.11) | .768 |
| 1.95 (1.07-3.56) | .028 | White blood cell count | 1.04 (0.95-1.14) | .429 |
| 1.97 (1.08-3.59) | .026 | Platelets | 1.00 (1.00-1.00) | .759 |
| 1.96 (1.08-3.58) | .027 | Albumins | 1.27 (0.83-1.95) | .264 |
| 1.94 (1.07-3.54) | .030 | Bilirubin | 1.02 (0.98-1.06) | .361 |
| 1.96 (1.08-3.57) | .027 | Creatinine | 0.99 (0.61-1.60) | .961 |
| 1.98 (1.09-3.61) | .025 | International normalized ratio | 1.07 (0.87-1.31) | .516 |
| 1.99 (1.09-3.65) | .026 | C-reactive protein | 0.97 (0.95-1.00) | .043 |
| 2.00 (1.10-3.66) | .024 | Cold ischemic time | 1.07 (0.93-1.24) | .358 |
| 1.97 (1.08-3.60) | .026 | Warm ischemic time | 1.01 (0.95-1.09) | .722 |
| 1.89 (1.03-3.45) | .039 | Intraoperative PRBC transfusions | 1.05 (0.98-1.14) | .170 |
| 1.93 (1.05-3.53) | .034 | Intraoperative FFP transfusions | 1.02 (0.94-1.10) | .689 |
| 1.96 (1.08-3.56) | .028 | Intraoperative dialysis | 1.30 (0.28-6.09) | .743 |
| 1.97 (1.08-3.59) | .026 | Donor age | 0.90 (0.73-1.12) | .357 |
| 1.95 (1.07-3.54) | .029 | Male donor sex | 0.78 (0.43-1.44) | .432 |
|  |  | Laboratory parameters after  intervention |  |  |
| 0.56 (0.17-1.80) | .327 | PaO_2_ | 1.07 (1.02-1.12) | .010 |
| 2.07 (1.13-3.80) | .018 | PaCO_2_ | 0.74 (0.47-1.15) | .176 |
| 2.07 (1.13-3.80) | .018 | pH | 1.00 (0.38-2.60) | .996 |
| 1.83 (0.90-3.72) | .094 | oxygen saturation | 1.08 (0.86-1.35) | .533 |
| 1.97 (1.07-3.62) | .029 | lactate concentration | 1.10 (0.99-1.23) | .073 |
| a – the estimates of coefficients were not presented due to the overestimation resulting from the small number of events. FiO_2_ – fraction of inspired oxygen; OR – odds ratio; 95% CI – 95% confidence interval; MELD – model for end–stage liver disease; HCV – hepatitis C virus; HBV – hepatitis B virus; ASA – American Society of Anesthesiologists; SENIC – Study on the Efficacy of Nosocomial Infection Control; NNISS – National Nosocomial Infections Surveillance System; PRBC – packed red blood cells; FFP – fresh frozen plasma; PaO_2_ – arterial partial oxygen pressure; PaCO_2_ – arterial partial carbon dioxide pressure. Odds ratios were calculated per 10 years increase for patient and donor age; 1 kg/m^2^ increase for BMI; 1 point increase for MELD; 1 hour increase for operative time and cold ischemic time; 1 g/dL increase for hemoglobin; 10^3^/cm^3^ increase for white blood count; 100 x 10^3^/cm^3^ increase for platelets; 1 g/dL increase for albumins; 1 mg/dL increase for bilirubin and creatinine; 1 increase for international normalized ratio; 1 mg/L increase for C–reactive protein; 10 min increase for warm ischemic time; 1 unit increase for PRBC and FFP transfusions; 10 mmHg increase for PaO_2_ and PaCO_2_; 1 increase for pH; 1% increase for oxygen saturation; 1 mmol/L increase for lactates. | | | | |
